# Antifibrotic and Regenerative Effects of Treamid in Pulmonary Fibrosis

**DOI:** 10.3390/ijms21218380

**Published:** 2020-11-08

**Authors:** Evgenii Skurikhin, Vladimir Nebolsin, Darius Widera, Natalia Ermakova, Olga Pershina, Angelina Pakhomova, Vyacheslav Krupin, Edgar Pan, Mariia Zhukova, Fedor Novikov, Lubov Sandrikina, Sergey Morozov, Aslan Kubatiev, Alexander Dygai

**Affiliations:** 1 Laboratory of Regenerative Pharmacology, Goldberg ED Research Institute of Pharmacology and Regenerative Medicine, Tomsk National Research Medical Centre of the Russian Academy of Sciences, Lenin, 3, 634028 Tomsk, Russia; nejela@mail.ru (N.E.); ovpershina@gmail.com (O.P.); angelinapakhomova2011@gmail.com (A.P.); vakrupin88@gmail.com (V.K.); artifexpan@gmail.com (E.P.); ermolaeva_la@mail.ru (L.S.); amdygay@gmail.com (A.D.); 2 “PHARMENTERPRISES” Ltd., 143026 Moscow, Russia; nve1970@mail.ru (V.N.); fnovikov@pharmenterprises.ru (F.N.); 3 Stem Cell Biology and Regenerative Medicine Group, School of Pharmacy, University of Reading, Whiteknights campus, Reading RG6 6AP, UK; d.widera@reading.ac.uk; 4 Siberian State Medical University, 634028 Tomsk, Russia; mashazyk@gmail.com; 5 Institute of General Pathology and Pathophysiology, 125315 Moscow, Russia; biopharm@list.ru (S.M.); niiopp@mail.ru (A.K.)

**Keywords:** pulmonary fibrosis, Treamid, regeneration, endothelial progenitor cells

## Abstract

Idiopathic pulmonary fibrosis (IPF) is a chronic progressive disease characterized by interstitial fibrosis and progressive respiratory failure. Pirfenidone and nintedanib slow down but do not stop the progression of IPF. Thus, new compounds with high antifibrotic activity and simultaneously regenerative activity are an unmet clinical need. Recently, we showed that Treamid can help restoring the pancreas and testicular tissue in mice with metabolic disorders. We hypothesized that Treamid may be effective in antifibrotic therapy and regeneration of damaged lung tissue in pulmonary fibrosis. In this study, experiments were performed on male C57BL/6 mice with bleomycin-induced pulmonary fibrosis. We applied histological and immunohistochemical methods, ELISA, and assessed the expression of markers of endothelial and epithelial cells in primary cultures of CD31^+^ and CD326^+^ lung cells. Finally, we evaluated esterase activity and apoptosis of lung cells in vitro. Our data indicate that Treamid exhibits antifibrotic activity in mice with pulmonary fibrosis and has a positive effect on capillaries of the lungs. Treamid also increases the number of endothelial progenitor cells in the lungs of animals with pulmonary fibrosis. Lastly, Treamid increases esterase activity and decreases apoptosis of CD31^+^ lung cells in vitro. Based on these findings, we suggest that Treamid may represent a promising compound for the development of new antifibrotic agents, which are capable of stimulating regeneration of lung endothelium in IPF patients.

## 1. Introduction

According to respiratory societies, the problem of treating idiopathic pulmonary fibrosis (IPF) is far from being solved [1,2]. Drugs widely used for the treatment of IPF (pirfenidone and nintedanib) do not show sufficient antifibrotic activity. Moreover, they only slow down but do not stop the progression of the disease [3] and are associated with severe side effects after long-term prescription [4,5,6]. The lack of effective treatment for IPF is aggravated by the fact that progressive pulmonary fibrosis is one of the main and serious complications in patients who have undergone COVID-19 coronavirus infection [7,8].

IPF is associated with both a vascular injury and a repair defect. The mechanisms that underlie these vascular abnormalities are still largely unknown. An important role of endothelial progenitor cells in lung repair has been suggested in animal models and in the human system [9,10]. It is suggested that endothelial cells may contribute to fibrosis in two ways: by becoming dysfunctional and by differentiating into highly fibrogenic mesenchymal cells referred to as myofibroblasts [11]. Current data suggest that the effect on endothelial dysfunction may potentially contribute to regeneration and recovery of the damaged pulmonary endothelium at the pulmonary fibrosis [9].

New metal chelators have been identified as effective therapeutic agents in regeneration of organs affected by diabetic degeneration [12,13,14,15]. Treamid (or bisamide derivative of dicarboxylic acid) is an effective complexing agent, for which high logK1 ≥ 5 (5.50 and 5.24) values were found in relation to transition metal ions [16]. Recently, we have shown that Treamid can chelate metal ions including zinc, copper, iron, magnesium, and calcium [16,17]. Treamid has regenerative properties and restores the function of various tissues and organs [16,17,18].

The aim of this study was to evaluate the antifibrotic and regenerative effects of Treamid in C57BL/6 mice with bleomycin (BLM)-induced pulmonary fibrosis. In addition, we evaluated the effect of Treamid on its potential cellular targets such as lung endothelial progenitor cells.

## 2. Results

### 2.1. The Effect of Treamid on Lungs Damaged by Bleomycin

#### 2.1.1. Effects of Treamid on Tissue Morphology

Bleomycin, a chemotherapeutic agent, leads to interstitial lymphocytic inflammation, alveolar epithelial cell injury, fibroblast proliferation, interstitial widening, and fibrosis. As an initial assessment, histological analysis of hematoxylin and eosin (HE)-stained lung sections was performed to examine whether the fibrotic response in mice receiving BLM was ameliorated by Treamid administration. Histological data obtained on d8 of BLM administration showed that BLM caused interstitial and interalveolar edema, hemorrhages, fibrin accumulation in the alveoli in group 2 mice compared to group 1 mice (Supplementary Materials Figure S1). The interstitial inflammatory infiltrate in the interalveolar septa consisted of lymphocytes, plasma cells, and macrophages. Fibrin thrombi of various degrees of organization were found in the capillaries of the walls of the alveoli and in the small branches of the pulmonary artery.

**Figure 1:**
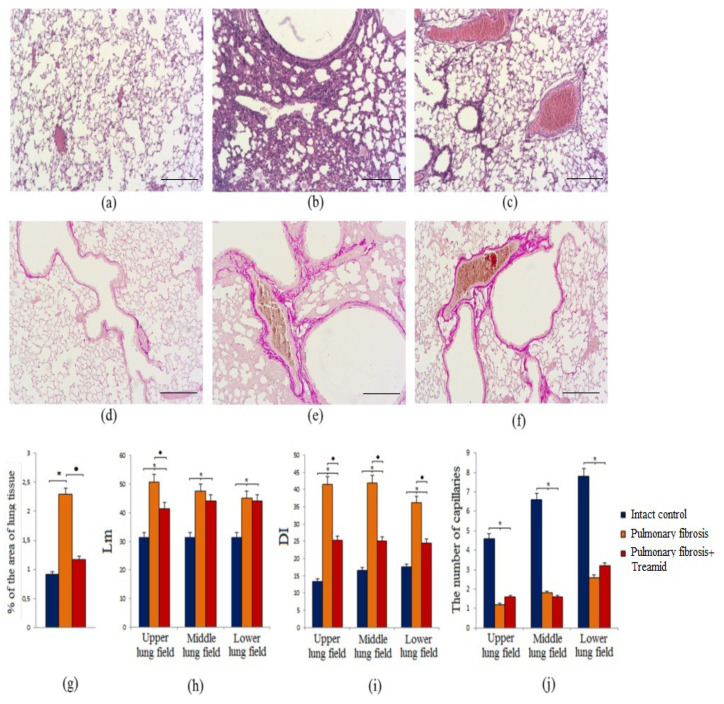
Photomicrographs of left lung sections (middle pulmonary field) obtained from male C57BL/6 mice on 21st day after the BLM injection (d21). (**a**,**d**) Mice of Intact control; (**b**,**e**) mice with pulmonary fibrosis; (**c**,**f**) mice with pulmonary fibrosis treated Treamid. (**a**–**c**) Tissues stained with hematoxylin-eosin; (**d**–**f**) tissues stained by Van Gieson, scale bar 100 μm. (**g**) Content of the connective tissue in the lungs of C57BL/6 mice at the pulmonary fibrosis on d21. (**h**) The mean linear intercept (Lm) in the lungs of male C57BL/6 mice with pulmonary fibrosis on d21 (upper, middle and lower pulmonary field). (**i**) Destructive index (DI) in the lungs of C57BL/6 mice with pulmonary fibrosis on d21 (upper, middle and lower pulmonary field). (**j**) The number of capillaries in five consecutive fields of lungs sections (upper, middle and lower pulmonary field) obtained from male C57BL/6 mice on d21. Groups: intact control; mice with pulmonary fibrosis (pulmonary fibrosis); mice with pulmonary fibrosis treated Treamid (pulmonary fibrosis+Treamid). * *p* < 0.05 significance of difference compared with intact control group, ● *p* < 0.05 significance of difference compared with the pulmonary fibrosis group.

On 21st day (d21) after the BLM injection (the fibrotic phase of pulmonary fibrosis after BLM treatment), infiltration of inflammatory cells (lymphocytes, macrophages) into the lung tissue was observed in group 2 mice (mice with BLM) (Figure 1a,b).

The inflammatory response has been found to be predominantly perivascular and peribronchial. In group 2 mice, fibrosis around the alveolar passages, collapse of the alveoli, an increase in the number of alveolocytes, and thickening of the interalveolar septa were found. In some areas of the lungs, restructuring of the lung tissue with the formation of a “honeycomb lung” was detected. In addition, BLM caused thickening of the walls of the alveoli and vascular congestion in the lungs.

Treamid was administered at d14 to assess the effect of the compound on the fibrotic phase of BLM model mice. Treamid reduced the density of lympho-macrophage infiltration of the lung parenchyma and the thickness of the alveolar walls in group 3 mice with (pulmonary fibrosis treated with Treamid) compared to mice in group 2 (d21). The treatment did not reveal vascular congestion and “honeycomb lung” in group 3 (Figure 1c). Notably, the destruction of the alveoli was less pronounced than in the untreated mice. Overall, administration of Treamid (10 mg/kg on d14–d20) ameliorated the lesions.

#### 2.1.2. Evaluation of Collagen Fibers

Van Gieson staining of lung preparations with picrofuchsin revealed deposition of collagen fibers in the lungs of group 2 mice (*p* < 0.05) which was absent in group 1 mice (d21). Deposition of collagen fibers was more profound adjacent to vessels and bronchioles (Figure 1e). Treamid reduced (*p* < 0.05) the size of collagen deposition in the alveolar walls of group 3 mice compared to group 2 mice (Figure 1f). Moreover, treatment with Treamid led to a decrease in the thickness of the alveolar septa and restoration of the airiness of the lung tissue.

#### 2.1.3. Mean Linear Intercept (Lm)

Mean linear intercept (Lm) measures are routinely used in the assessment of clinically relevant lung pathology [19]. BLM significantly increased (*p* < 0.05) Lm in the upper, middle, and lower lung segments in group 2 mice compared to group 1 mice (d21) (Figure 1h). Treamid had no effect on Lm in the middle and lower lungs of group 3 mice compared to group 2 mice. Finally, Treamid (10 mg/kg on d14–d20) decreased (*p* < 0.05) Lm in the upper segment of the lungs (Figure 1h).

#### 2.1.4. Destructive Index (DI)

The degree of morphologic changes in affected lung may be assessed by stratified random field selection and subsequent qualitative morphometric and pathologic analysis, such as the DI [20]. Modeling of pulmonary fibrosis caused a significant increase (*p* < 0.05) of the DI in the upper, middle and lower lung segments of group 2 mice compared to group 1 mice (d21) (Figure 1i). Treamid reduced (*p* < 0.05) DI in all lung segments in group 3 mice compared to group 2 mice (Figure 1i).

#### 2.1.5. Capillaries of the Lungs

Altered vascular architecture occurred predominantly in the subpleural and peribronchial regions in BLM-treated mice, whereas the vehicle group showed intact lung architecture. BLM administration reduced (*p* < 0.05) the number of capillaries in the lungs of group 2 mice compared to group 1 mice (d21) (Figure 1j). Treamid caused a consistent trend towards an increase in the number of capillaries in the upper and lower lungs of group 3 mice compared to group 2 mice (Figure 1j). Moreover, the microvascular architecture in the lungs of Treamid-treated mice resembled normal, autochthonous vascular lung architecture with alveolar plexus.

### 2.2. Effect of Treamid on the Molecules of Fibroblastic Process Expression in Bleomycin Damaged Lungs

**Figure 2:**
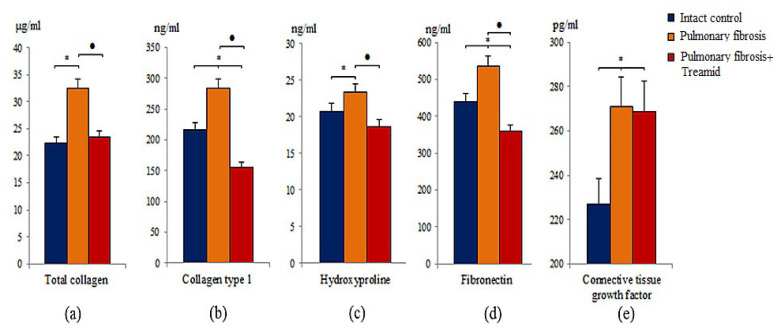
Effects of Treamid treatment on total soluble collagen (**a**), collagen type I (**b**), hydroxyproline (**c**), fibronectin (**d**), and connective tissue growth factor (**e**) levels in homogenate of right lung lobes received from male C57BL/6 mice (d21). Groups: intact control; mice with pulmonary fibrosis (pulmonary fibrosis); mice with pulmonary fibrosis treated Treamid (pulmonary fibrosis+Treamid). * *p* < 0.05 significance of difference compared with intact control group, ● *p* < 0.05 significance of difference compared with the pulmonary fibrosis group.

In mice with pulmonary fibrosis, an increase of total collagen, type 1 collagen, hydroxyproline, fibronectin, and connective tissue growth factor (*p* < 0.05) was observed in the lung homogenates of group 2 mice compared to group 1 mice on d21 (Figure 2). After Treamid administration (10 mg/kg on d14–d20), lower (*p* < 0.05) levels of total collagen, type 1 collagen, hydroxyproline, and fibronectin were found in lung homogenates of group 3 mice than in group 2 mice (d21) (Figure 2). The treatment did not affect connective tissue growth factor.

### 2.3. Effects of Treamid on IL-13 in Bleomycin Damaged Lungs

IL-13 has been shown to contribute to inflammation and fibrosis in multiple animal models of pulmonary fibrosis including bleomycin-induced pulmonary fibrosis [21]. Pulmonary fibrosis caused an increase in the level of IL-13 in the lung homogenate of group 2 mice compared to group 1 mice (d21) (Supplementary Materials Figure S2). After Treamid administration, significantly lower (*p* < 0.05) levels of IL-13 were found in lung homogenates of group 3 mice compared to group 2 mice (Supplementary Materials Figure S2).

### 2.4. Effects of Treamid on Lung Endothelial Progenitor Cells In Vivo

**Figure 3:**
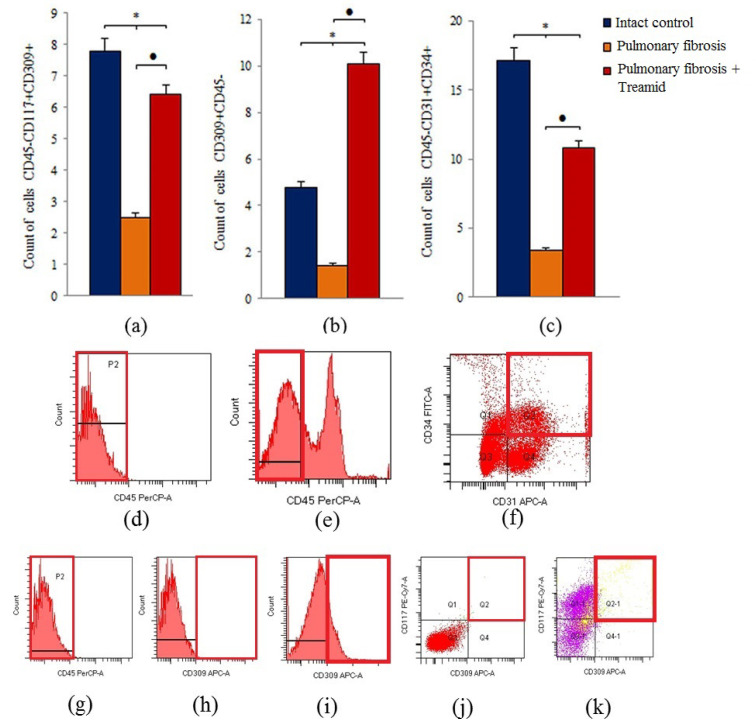
Characterization of CD45^−^CD117^+^CD309^+^, CD309^+^CD45^−^, CD45^−^CD31^+^CD34^+^ endothelial cell population isolated from lung of male C57BL/6 mice on d21. Cells were analyzed by flow cytometry using antibodies for mouse CD45, CD31, CD34, CD117, CD309. Dot plots are representative for three independent experiments with the mean from three independent experiments. (**a**) The content of angiogenesis progenitors (CD45^−^CD117^+^CD309^+^) in the lung of mice. (**b**) The content of endothelial cells (CD309^+^CD45^−^) in the lung of mice. (**c**) The content of endothelial progenitor cells with phenotype CD45^−^CD31^+^CD34^+^ in the lung of mice. Groups: control, pulmonary fibrosis and pulmonary fibrosis treated by Treamid. * Significance of difference compared with intact control (*p* < 0.05); ● significance of difference compared with the pulmonary fibrosis group (*p* < 0.05). (**d**) Isotype control for IgG2a (PerCP). (**e**) Phenotype establishment and qualitative analysis of CD45 (PerCP). (**f**) Phenotype establishment and qualitative analysis of CD34 (FITC), CD31 (APC). (**g**) Isotype control for IgG2a (PerCP). (**h**) Isotype control for IgG2b (APC). (**i**) Phenotype establishment and qualitative analysis of CD309 (APC) expression. (**j**) Isotype control for IgG2b (PE-Cy7) and for IgG2b (APC). (**k**) Phenotype establishment and qualitative analysis of CD117 and CD309 (APC) expression.

To identify the mechanism underlying the antifibrotic and regenerative effects of Treamid, flow cytometry was used to measure the number of lung endothelial progenitor cells in the lung tissue. Pulmonary fibrosis decreased (*p* < 0.05) the number of hemangiogenesis precursors (CD45^−^CD117^+^CD309^+^), endothelial progenitor cells (CD45^−^CD31^+^CD34^+^) VEGF2^+^ endothelial cells (CD309^+^CD45^−^), epithelial cells (CD45^−^Ter119^−^CD326^+^), and Clara cells (CD45^−^CD34^−^CD31^−^Sca1^+^) in the lungs of group 2 mice compared to group 1 mice (d21) (Figure 3, Supplementary Materials Figure S3). In addition, there was a tendency towards a decrease in the number of epithelial progenitor cells (CD45^−^CD117^+^CD49f^+^) (0.460 ± 0.044—control and 0.400 ± 0.005—mice with pulmonary fibrosis). Treamid administration caused a significant increase (*p* < 0.05) in the number of hemangiogenesis precursors, endothelial progenitor cells, and VEGF2^+^ endothelial cells in the lungs of group 3 mice compared to group 2 mice (Figure 3). Treamid increased (*p* < 0.05) the number of Clara cells in the lungs but had no effect on epithelial progenitor cells.

### 2.5. The Effect of Treamid on CD31^+^ Cells In Vitro

**Figure 4:**
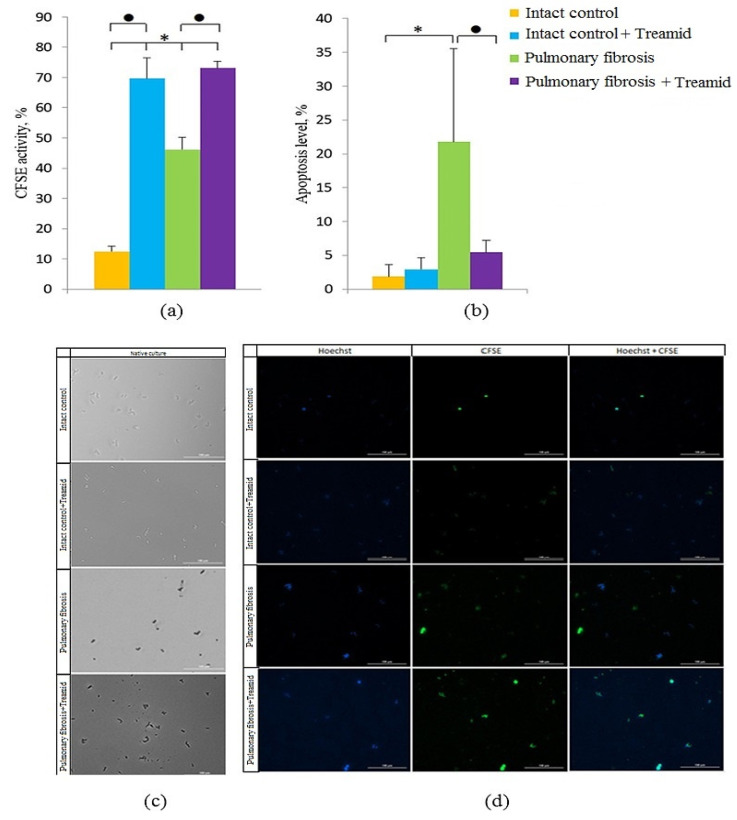
Effects of Treamid on culture of CD31^+^ endothelial cells isolated from the lungs of male C57BL/6 mice. (**a**) The level of carboxyfluorescein succinimidyl ester (CFSE) activity and (**b**) the count of apoptotic CD31^+^ cells after culture with or without Treamid (10^−7^ M). All data are expressed as mean±SD, * significance of difference compared with control (*p* < 0.05); ● significance of difference compared without Treamid group (*p* < 0.05). (**c**) CD31^+^ endothelial cells were precultured for five days, and incubated with or without Treamid (10^−7^ M) for 1 h; (**d**) culture of CD31^+^ endothelial cells from lung were precultured for 5 days, and incubated with or without Treamid (10^−7^ M) for 1 h and then labeled with Hoechst and CFSE (FITC) prior to fluorescence microscopic analysis. Images (20×) of CD31^+^ cells stained with: Hoechst (blue) to identify cell nuclei; CFSE (green); (Hoechst + CFSE) composite image using all two colors. Determination of the percentage of cells with CFSE activity was made as a ratio of cells counted in the green channel to total positive cells. All scale bars are 100 µm.

Angiogenesis is a physiological process that maintains organ homeostasis. In contrast, dysregulated neovascularization is involved in pathological conditions such as pulmonary fibrosis. We observed an increase in the number of apoptotic CD31^+^ cells in culture of cells isolated from mice with pulmonary fibrosis compared to cells isolated from group 1 mice (Figure 4b). The effects of Treamid on CD31^+^ cells were studied on cells isolated from lungs of groups 1 and 3. We found that Treamid significantly reduced the number of apoptotic CD31^+^ cells in group 3 (Figure 4b, 3.9 times *p* < 0.05) but had no significant effects on the number of apoptotic CD31 ^+^ cells in group 1. Treamid increased the number of CD31^+^ cells with active esterases in group 3 (by 58% compared to control culture, *p* < 0.05) (Figure 4a). A similar effect was observed in group 1.

## 3. Discussion

IPF is a chronic progressive disease characterized by a development of interstitial fibrosis, and progressive respiratory failure [22,23]. In most cases, the prognosis of IPF is unfavorable, with a life expectancy of five years after diagnosis [24,25]. Risk factors that can precipitate the development of fibrotic disease are hereditary factors, repeated exposure to toxins, smoking, chronic autoimmune inflammation, myocardial infarction, high serum cholesterol, obesity, diabetic disorders, and hypertension [23,26]. In addition, inflammation can be significant in the initiation and progression of fibrosis [27,28]. Generally, chronic inflammation, which lasts for months or years, precedes the nascence of IPF.

In IPF, the main cellular targets are alveolar epithelial cells. In this study, we demonstrated that BLM disrupts the integrity of the alveoli and damages alveolar epithelial cells (Figure 1, Supplementary Materials Figure S4). Moreover, we observed an increase in the numbers of mature epithelial cells (CD45^−^Ter119^−^CD326^+^) and epithelial progenitor cells (CD45^−^Ter119^−^CD49f^+^) in lungs of mice of group 2 (Supplementary Materials Figure S3). This discrepancy between the in vitro and in vivo experiments could be explained by inhibitory effects of inflammation on epithelial cells. The cells that contribute to this phenomenon might by interstitial macrophages migrating into the lungs after BLM administration (Supplementary Materials Figure S5). This is in general accordance with previous studies showing a recruitment of interstitial macrophages from the circulation into the lungs in response to acute lung injury [21,23].

Various independent research groups have shown that in pulmonary fibrosis, damaged cells of the alveolar epithelium are able to induce the migration, proliferation and activation of mesenchymal cells, which are involved in the fibroplastic process [27,28,29]. In this study, we have found an accumulation of MSCs (CD45^‒^CD31^‒^CD34^‒^CD73^+^CD90^+^) in the lungs of mice treated with BLM (Supplementary Materials Figure S6). In this population of MSCs, the proportion of cells expressing Notch1 significantly increased (Supplementary Materials Figure S6). In addition to these changes, group 2 mice showed an accumulation of fibroblasts in the lungs and the formation of fibroblasts foci (Figure 1). Using ELISA, we demonstrated upregulation of total collagen, type 1 collagen, hydroxyproline, fibronectin, and connective tissue growth factor in lung homogenates from group 2 (Figure 2). Moreover, we were able to show deposition of collagen fibers which might be a result of an activation of the fibroplastic process. Notably, this process was most intense around the vessels and bronchioles.

The endothelium plays a central role in pulmonary vascular regulation and endothelial dysfunction and is increasingly viewed as pivotal for initiation and progression of IPF [30]. In IPF, a pathological vascular remodeling is observed [9,10,31,32]. Loss of the normal structure and function of the endothelium during the destruction of epithelial cells can irreversibly lead to the loss of alveolar-capillary integrity, after which the progression of fibrosis is difficult to stop. In areas of the lungs with fibrosis, we found few or no blood vessels compared to healthy lungs, and a decreased CD31 expression in the alveoli (Figure 1, Supplementary Materials Figure S7). In the lung tissue of mice with pulmonary fibrosis, we found a significant decrease in the number of endothelial progenitor cells (CD45^−^CD31^+^CD34^+^), VEGF2^+^ cells (CD309^+^CD45^−^), and hemangiogenesis precursors (CD45^−^CD117^+^CD309^+^) (Figure 3). At the same time, CD31^+^ cells of group 2 had a decreased esterase activity and increased frequency of apoptosis compared to group 1 in vitro (Figure 4).

After treatment with Treamid, we observed a decrease in lung inflammation in group 3 mice compared to group 2 mice as evidenced by a decrease in the density of lympho-macrophage infiltration of the lung parenchyma (Figure 1), and a decrease in CD16 expression in the interstitium and lung parenchyma (Supplementary Materials Figure S7). Cytometric analysis revealed a reduction in the number of mononuclear phagocytes of various phenotypes in the lungs: CD11b^+^ (total population of mononuclear phagocytes), CD45^+^CD19^−^Sca1^+^CD11b^−^F4/80^+^ (alveolar macrophages), CD45^+^CD90^+^CD44^+^ (circulating monocytes and lymphocytes) (Supplementary Materials Figure S8). As IL-13 is well-known, to induce respiratory disease [21,33,34], the decrease of the IL-13 concentration in the lung homogenate in group 3 mice (Supplementary Materials Figure S2) might represent an additional mechanistic aspect of the regenerative effects of Treamid. IL-13 is known to activate fibroblasts that participate in the production and deposition of excess collagen and fibronectin in the lungs and other organs [21]. However, anti-IL-13 clinical trials were stopped due to lack of efficacy and could not meet their primary end points. This suggests that targeting the IL-13 alone may not be beneficial in IPF.

Treamid reduced collagen deposition in the alveolar walls of group 3 mice compared to group 2 mice. This could be explained by the anti-inflammatory effects of Treamid. In contrast to group 2 mice, “honeycomb lungs” were not found in the treated mice. In addition, Treamid might interfere with collagen production. This hypothesis is supported by much lower levels of total collagen, type 1 collagen, hydroxyproline and fibronectin in group 3 mice than in group 2 mice (Figure 2).

Treamid-mediated reduction of the inflammation and fibrosis had positive effects on the condition of the alveoli. We found a decrease in alveolar destruction and a decrease in their wall thickness in the treated mice. This led to the restoration of the alveoli structure of the lung tissue. However, from our point of view, the most important effect of Treamid is the observed improvement of the lung microvasculature. Lungs of group 3 mice did not reveal vascular congestion similar to that in group 2 (Figure 1). Moreover, Treamid caused an increase in the number of capillaries in the upper and lower lungs (Figure 1), and an increased expression of VEGF2 (Figure 3). Our findings suggest that pulmonary fibrogenesis and angiogenesis interact in the progression of pulmonary fibrosis. Treamid might interfere with these pivotal pathogenetic mechanisms in pulmonary fibrosis, significantly improving lung function and normalizing the microvascular architecture.

Overall, the beneficial effects of Treamid could be explained by its action on precursors of endothelial cells. As shown in Figure 3, Treamid significantly increased the number of hemangiogenesis precursors (CD45^−^CD117^+^CD309^+^), endothelial progenitor cells (CD45^−^CD31^+^ CD34^+^), and VEGF2^+^ endothelial cells in the lungs of group 3 mice compared to group 2 mice. In vitro, Treamid had no significant effect on CD31^+^ lung cells from group 1 mice (Figure 4). In contrast, Treamid decreased the number of apoptotic cells and increased the number of cells with active esterases in the culture of CD31^+^ lung cells from group of mice with pulmonary fibrosis (Figure 4). Thus, we associate the revealed regenerative effect of Treamid on the pulmonary fibrosis model with the precursors of endothelial cells with CD31^+^ being potential cellular targets. These findings are supported by the fact that Treamid does not affect Clara cells and epithelial progenitor cells (CD45^−^CD117^+^CD49f^+^) in vivo and in vitro (Supplementary Materials Figures S3 and S4).

## 4. Materials and Methods

### 4.1. Animals

Nine-week-old male C57BL/6 mice (Surgical Bio-modelling Department of the Goldberg ED Research Institute of Pharmacology and Regenerative Medicine, Tomsk, Russia) were used in all experiments. Animals were randomly assigned into the experimental groups. All experimental protocols were approved by the animal care and use committee of the Goldberg ED Research Institute of Pharmacology and Regenerative Medicine, Tomsk NRMC (IACUC Protocol No. 71052014, 30.09.2016). Within this study, 60 mice were used.

### 4.2. Modeling of Experimental Pulmonary Fibrosis

Experimental pulmonary fibrosis was induced by a single intratracheal bleomycin (BLM, Nippon Kayaku Co., Ltd., Tokyo, Japan) administration at a dose 80 μg/mouse in 0.03 mL of 0.9% NaCl, which was slowly instilled in the tracheal lumen [35]. All procedures were performed under anesthesia induced by intraperitoneal injection of chloral hydrate (400 mg/kg intraperitoneal). These animals formed the BLM control. Control animals were administered a single intratracheal 0.03 mL 0.9% NaCl. The introduction of BLM was defined as day zero (d0). All mice were euthanized on d21 by CO_2_.

Mice were cohoused (five to six mice per cage) and entrained to a reverse 12 h light/12 h dark cycle. Throughout the experimental period, mice had ad libitum access to standard rodent chow.

### 4.3. Treamid

**Figure 5:**
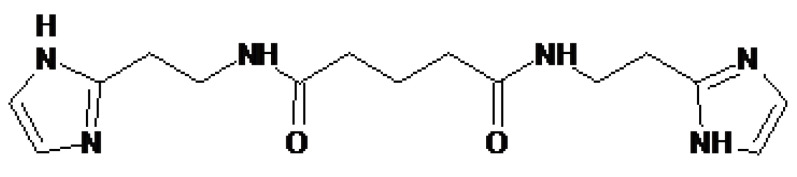
Structural formula of pharmacological compound Treamid, synonym bisamide derivative of dicarboxylic acid, chemical formula N1, N5-bis [2-(1H-imidazole-2-Il)ethyl] glutaramide.

Treamid (bisamide derivative of dicarboxylic acid (BDDA), also known as XC268BG), chemical formula N1,N5-bis [2-(1H-imidazole-2-Il)ethyl] glutaramide, was provided by the General Director of the “PHARMENTERPRISES” Ltd. Mr. V. Nebolsin (Figure 5) [16,17,18].

Treamid was dissolved in 0.5% carboxymethylcellulose solution (vehicle) and was administered intragastrically daily at a dose of 10 mg/kg on d14–d20. The volume of administration was determined according to body weight. The Treamid dose was selected according to previous reports [16,17,18]. Treamid was administered in a therapeutic setting beginning at day 14 (d14) to assess the effect of the drug on the fibrotic phase of BLM model mice. Control animals received the solvent in the equivalent volume.

Treamid was used at a concentration of 10^−7^ M in vitro as described earlier [18], where Treamid was titrated to determine the optimal concentration in culture.

### 4.4. Experimental Groups

Animals were allocated into three groups (n = 20/group): intact control (mice without BLM receiving saline solution formed the control group—group 1), pulmonary fibrosis (mice with BLM—group 2), and pulmonary fibrosis + Treamid (group 3—pulmonary fibrosis with Treamid treatment).

**Figure 6:**
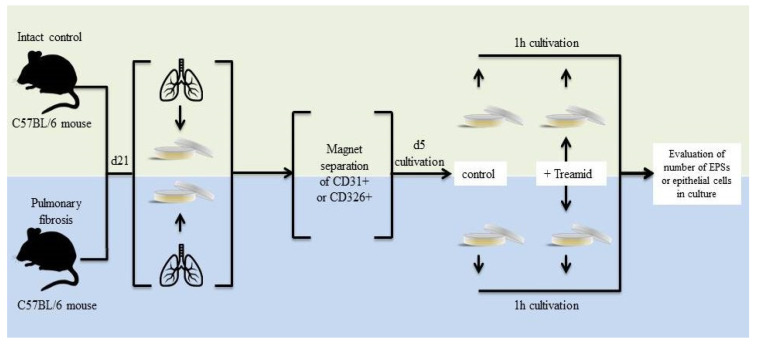
Graphical scheme of the protocol for CD31^+^ lung endothelial cells and CD326^+^ lung epithelial cells cultivation with Treamid.

Direct effects of Treamid on endothelial cells and epithelial cells were investigated in cultivated mononuclear cells isolated from healthy mice and mice with pulmonary fibrosis on d21 (Figure 6).

For the in vitro experiments using CD31^+^ cells, four experimental groups were used: group 1—control (CD31^+^cells isolated from intact mice after culture), group 2—intact control + Treamid (CD31^+^cells isolated from intact mice after culture + Treamid (10^−7^ M) for 1 h), group 3—pulmonary fibrosis (CD31^+^ cells isolated from mice with pulmonary fibrosis after culture), group 4—pulmonary fibrosis + Treamid (CD31^+^ cells isolated from mice with pulmonary fibrosis after culture + Treamid (10^−7^ M) for 1 h) (Figure 6).

For the in vitro experiments using CD326^+^ cells, the following experimental groups were used: group 1—control (CD326^+^cells isolated from intact mice after culture), group 2—intact control + Treamid (CD326^+^ cells isolated from intact mice after culture + Treamid (10^−7^ M) for 1 h), group 3—pulmonary fibrosis (CD326^+^ cells isolated from mice with pulmonary fibrosis after culture), group 4—pulmonary fibrosis + Treamid (CD326^+^ cells isolated from mice with pulmonary fibrosis after culture + Treamid (10^−7^ M) for 1 h) (Figure 6).

### 4.5. Enzyme-Linked Immunosorbent Assay

#### 4.5.1. Hydroxyproline, Collagen Type I, Fibronectin, and Connective Tissue Growth Factor Measurements

Hydroxyproline and collagen type I, fibronectin, and connective tissue growth factor (CTGF) were quantified in homogenate of right lung lobes. Hydroxyproline, collagen type I, fibronectin, and CTGF were determined by ELISA according to manufacturer instructions (Cusabio Biotech CO., Ltd., Wuhan, China). The right lung lobes were excised and snap frozen after having measured the wet weight. Sensitivities were >1.95 ng/mL for hydroxyproline and CTGF, >0.039 ng/mL for collagen type I, >0.78 ng/mL for fibronectin.

#### 4.5.2. Total Soluble Collagen Assay

Total soluble collagen was quantified in homogenate of right lung lobes. The right lung lobes homogenate supernatants were placed in 1.5 mL tubes. Sircol-dye was added, the content of the tubes homogenized for 30 min and centrifuged for 10 min (10.000× *g*). Pellets were dissolved with alkaline reagent. Absorbance was read at 540 nm. The total soluble collagen was determined using a standard curve for the SircolTM assay (S1000, Biocolor Ltd., Country Antrim, UK) according to the manufacturer’s instructions. Results were expressed as mg collagen per right lung. Detection limit: 1.0 µg.

#### 4.5.3. Interleukin 13 Measurements

Interleukin 13 was measured in lung homogenates using specific ELISA kit from Cusabio according to manufacturer instruction (Cusabio Biotech CO., Ltd., Wuhan, China). The right lung lobes were excised and snap frozen after having measured the wet weight. Sensitivity was >7.8 ng/mL.

### 4.6. Histological Examination of Lung Tissue

The morphological examination of lungs was performed on d21. For histological studies, left lung lobes were fixed in 4% neutral formalin solution, embedded in paraffin and sections with thickness of 5 µm were prepared. Subsequently, sections were staining with hematoxylin and eosin. Histological examination was carried out in three areas of lung tissue (upper, middle and lower) on d21 as described previously [36]. Lung structure, presence of edema, infiltration by proinflammatory cells as well as venous hyperemia, and vascular and bronchial walls thickening were assessed [37,38,39]. Micropreparations from each experimental animal were examined under the light microscope Axio Lab.A1 (Carl Zeiss, MicroImaging GmbH; Göttingen, Germany) at 100× and 400× magnifications.

The degree of alveolar septa destruction was determined by measuring the destructive index (DI), and increase of alveolar spaces was determined by calculating average linear interception Lm. Mean linear intercept (MLI or Lm) is a measure of morphometric change based on serial measurements of the lung using test lines but cannot be directly interpreted as a measure of alveolar size. MLI can be most directly interpreted as the mean free distance between gas exchange surfaces in the acinar airway complex [19]. Importantly, the use of Lm in the estimation of the structural substrate of lung function is only applicable when considered a function of the total volume and surface area of the lung due to the lung’s structural complexity and complex inflationary behavior. Lm calculation was performed using FIJI and a 500 × 500 µm grid (10 horizontal and 10 vertical equally spaced lines) was placed in a micrograph of a histological section lightly stained with hematoxylin-eosin. Lm was calculated using the formula [40,41,42]:

Lm = (N × L)/m

where N is the number of lines placed on the tissue, L is the length of one line, m is the sum of all intersections.

Pulmonary parenchyma destruction degree was determined as DI [43]. Briefly, a grid consisting of 50 points with an area of 500 × 500 µm was placed on a microphotograph of a lung tissue using a FIJI. The structures underlying these points were classified as normal or destroyed alveolar spaces or airways. The points falling onto the other structures such as the airways or alveoli walls were not considered. DI was calculated using the formula [43]:

DI = (D/(D + N)) × 100

Structures lying under these points were classified as normal (N) or destroyed (D) alveolar and/or duct spaces. Points falling over other structures, such as duct walls, alveolar walls, etc., were not considered.

Micropreparations from each experimental animal were stained with hematoxylin and eosin were examined under an Axio Lab.A1 microscope (Carl Zeiss, MicroImaging GmbH, Göttingen, Germany) at 2500-fold magnification using immersion microscopy to assess the microvasculature state. The number of capillaries in five consecutive fields of view was calculated for each section [42,44].

To count collagen fibers in lung parenchyma, the histological slides were stained by picrofuchsin using Van Gieson method [45,46]. At least 10 photomicrographs without overlapping across the cut surface of the lung tissue at 100 x magnification were taken for each experimental animal. The used system consisted of a microscope (Axio Lab.A1, Carl Zeiss MicroImaging GmbH; Göttingen, Germany) with a video camera (AxioCam ERc5s, Carl Zeiss; Göttingen, Germany), connected to a personal computer. Gathered images were processed using the software AxioVision Rel.4.8.2. The content of collagen fibers in lung was determined using a function for counting the area of the object in the image. Bronchovascular strands were carefully removed from the analyzed areas. To count collagen fibers was calculated using the formula:

X = Σ a × 100/(S ‒ Σ b)

where ∑ a is the sum of the pixels occupied by tissue with fibrosis in 10 images of one specimen, S is the number of pixels corresponding to the full area of the image (when using this camera and the program—4423680), b is the sum of pixels occupied by the empty part of the slide, in 10 images one drug [45,46].

### 4.7. Flow Cytometric Analysis

Mononuclear cells from lungs were isolated as described earlier [47,48] and the expression of surface markers on mononuclear cells derived from lungs was analyzed. Fc-receptors were blocked by preincubation of the cells with unconjugated anti-CD16/CD32 antibodies for 10 min (eBioscience, San Diego, CA, USA, Clone: 93, Cat# 14-0161-85, 1/50 dilution) in 50 μL of 0.1% saponin (Sigma-Aldrich, St. Louis, MO, USA, Cat# S4521) and 1% BSA (Sigma-Aldrich, St. Louis, MO, USA, Cat# A3059-100G) in phosphate buffered saline (PBS) per tube. After the preincubation, cells suspensions were stained with fluorophore-conjugated monoclonal antibodies: CD45 PerCP (QC Testing: Mouse, Clone: 30-F11, Cat# 557235, 1/100 dilution) or CD45 APC-Cy 7 (QC Testing: Mouse, Clone: 30-F11, Cat# 557659, 1/100 dilution), CD31 APC (QC Testing: Mouse, Clone: MEC 13.3, Cat# 551262, 1/50 dilution), CD34 FITC (QC Testing: Mouse, Clone: RAM34, Cat# 560238, 1/50 dilution), CD309 (Flk-1) APC (QC Testing: Mouse, Clone: Avas 12alpha1, Cat# 560070, 1/50 dilution), CD117 PeCy7(QC Testing: Mouse, Clone: 2B8, Cat# 558163, 1/50 dilution), Sca1 Per-Cy5.5 (QC Testing: Mouse, Clone: D7, Cat# 561021, 1/50), CD73 V450 (QC Testing: Mouse, Clone: TY/23, Cat# 561544, 1/50), CD44 APC (QC Testing: Mouse, Clone: IM7, Cat# 559250, 1/50), CD90 PerCP (QC Testing: Mouse, Clone: OX-7, Cat# 557266, 1/50), CD44 APC (QC Testing: Mouse, Clone: IM7, Cat# 559250, 1/50), TER119 APC-Cy 7 (QC Testing: Mouse, Clone: TER-119, Cat# 560509, 1/100), CD19 FITC (QC Testing: Mouse, Clone: D3, Cat# 553785, 1/50), CD11b Alexa Fluor^®^ 647 (QC Testing: Mouse, Clone: M1/70, Cat# 557686, 1/50), F4/80 BV421 (QC Testing: Mouse, Clone: T45-2342, Cat# 565411, 1/50), Notch1 PE (QC Testing: Mouse, Clone: mN1A, Cat# 552768, 1/50 dilution) (all Becton Dickinson, San Jose, CA, USA), and CD326 (EpCam) PE (QC Testing: Mouse, Clone: caa7-9G8, Cat# 130-102-265, 1/50 dilution, Miltenyi Biotec B.V. & Co. KG, Bergisch Gladbach, Germany). The antibody rat antihuman CD49f FITC (Clone: GoH3, Cat# 555735, 1/50, Becton Dickinson, San Jose, CA, USA) was rat antihuman with reactivity for mouse. All antibodies were titrated to determine their optimal staining concentration and appropriate isotype controls were used. Labeled cells were washed thoroughly with 500 μL of FACSFlow (Becton Dickenson, Franklin Lakes, NJ, USA, Cat# 342003).

For the intracellular staining of the Notch1, a Fixation/Permeabilization Solution Kit (Becton Dickinson, San Jose, CA, USA, Cat# 554714) was used in accordance with the manufacturer’s instructions. After cell fixation and permeabilization, the BD Perm/Wash™ Buffer was used to wash the cells and to dilute the Notch1 antibodies for staining.

All samples were run on a Becton Dickenson FACSCanto II flow cytometer. The instrument was set up and standardized using BD Cytometer Setup and Tracking (CS&T) procedures according to manufacturer specifications. One hundred thousand events per tube were acquired. Data were analyzed using FACSDiva 8.0 software.

### 4.8. Lung Tissue Dissociation, Isolation and Magnetic Separation of CD31^+^ Lung Endothelial Cells

On d21 we studied the effect of Treamid on CD31^+^ lung endothelial cells of mice of the control group (group 1) and mice with pulmonary fibrosis (group 2) in vitro. Lung endothelial cells were isolated using protocol [47,48,49].

All metal instruments were sterilized by autoclaving; sterile disposables were from BD Falcon. Lungs were isolated from surrounding tissue, rinsed with PBS (Sigma-Aldrich, St. Louis, MO, USA), mechanically dispersed, and minced with scissors. The digestion buffer containing 0.3 mg/mL Collagenase I type (Sigma-Aldrich, St. Louis, MO, USA) with PBS (Sigma-Aldrich, St. Louis, MO, USA) was used per lung on an orbital shaker at 37 °C for 6 h. The cells suspension was gently mixed using an 18-gauge needle, sequentially filtered through a 70 μm and 40 μm cell strainers (Falcon, Becton Dickinson) into fresh tube and washed twice in PBS with the addition of 2% fetal bovine serum (FBS, Sigma-Aldrich, St. Louis, MO, USA) by centrifugation at 900× *g* (4 min, 4 °C).

Magnetic sorting was performed using EasySep™ Mouse Biotin Positive Selection Kit (Catalog #18556, StemCell Technologies, Vancouver, BC, Canada) and Biotin Rat Anti-Mouse CD31 antibodies (QC Testing: Mouse, Clone: MEC 13.3, Cat# 553371, 1/50 dilution, Becton Dickinson, San Jose, CA, USA). The fraction of CD31^+^ cells was isolated using EasySep™ Magnet (StemCell Technologies, Vancouver, BC, Canada).

Assessment of the EasySep™ cell separation efficiency was carried out using flow cytometry as described above.

### 4.9. Cultivation of CD31^+^ Lung Endothelial Cells with Treamid

The fraction of CD31^+^ cells (10^6^ cells /1 mL of medium) obtained after magnetic sorting was cultured on collagen-coated plastic plates for T-25 cell cultures in the medium M199 (Sigma-Aldrich, St. Louis, MO, USA) consisted of 15% FBS (Sigma-Aldrich, St. Louis, MO, USA), 1 mM L-glutamine (Sigma-Aldrich, St. Louis, MO, USA), 100 μg/mL heparin (Sigma-Aldrich, St. Louis, MO, USA) and endothelial growth factor 50 μg/mL (Sigma-Aldrich, St. Louis, MO, USA) [47,50]. Cells were cultured for five days in standard gas (3.5% CO_2_) and temperature conditions (37° C). The medium was changed every one to two days.

After a five-day culture cycle, CD31^+^ cells of groups 1 and 2 were removed from the plate using a warm 0.05% trypsin-EDTA solution (Sigma-Aldrich, St. Louis, MO, USA), changed to a medium of the following composition: M199 with 15% FBS (Sigma-Aldrich, St. Louis, MO, USA), 1 mM L-glutamine (Sigma-Aldrich, St. Louis, MO, USA), 100 μg/mL of heparin (Sigma-Aldrich, St. Louis, MO, USA) and growth factor endothelium 50 μg/mL (Sigma-Aldrich, USA), and the cells were sown at a concentration of 3 × 10^5^ cells/1 mL of medium on collagen-coated plastic T-25 plates. Before cultivation on the medium with CD31^+^ cells, we added Treamid (10^−7^ M). The cultivation cycle under standard conditions (3.5% CO_2_, 37 °C) was 1 h. At the end of cultivation, the effectiveness of Treamid on Carboxyfluorescein succinimidyl ester (CFSE) and apoptosis levels in CD31^+^ cell culture was evaluated using image processing in each well of Cytation ™ 3.

### 4.10. Lung Tissue Dissociation, Isolation and Magnetic Separation of CD326^+^ Lung Epithelial Cells

On the d21 we studied the effect of Treamid on CD326^+^ epithelial cells isolated from lungs of mice from the control group (group 1) and mice with pulmonary fibrosis (group 2) in vitro. Lungs were isolated from surrounding tissue, rinsed with PBS, mechanically dispersed, and minced with scissors. We isolated lung cells as described above in Section 4.8. Lung epithelial cells were isolated using protocol [51,52]. The single cells suspension was gently mixed using an 18-gauge needle, sequentially filtered through a 70 μm cell strainer (Falcon, Becton Dickinson) and washed twice in PBS with the addition of 2% FBS (Sigma-Aldrich, St. Louis, MO, USA) and 0,1% BSA (Sigma-Aldrich, St. Louis, MO, USA) by centrifugation at 700× *g* (5 min, 4 °C).

Low-density cells were then isolated by density gradient centrifugation Histopaque-1077 (Sigma-Aldrich, St. Louis, MO, USA) in a 2:1 ratio (two suspensions per gradient) and then resuspended and centrifuged for 20 min at room temperature at 1500 g. Harvest low-density cells from the interface between the PBS layer and the Histopaque-1077 solution using a 10 mL pipet and transfer into a 50 mL centrifuge tube, washed twice in excess PBS supplemented with 0.1% BSA and 2% FBS 2% and centrifuged for 5 min at 700× *g* and 4 °C. Counted the cells and calculated cell concentration.

The resulting cell suspension was subjected to magnetic sorting to enrich CD326^+^ cells. Magnetic sorting was performed using EasySep™ Mouse Epithelial Cell Enrichment Kit II (Catalog # 19868, Immunomagnetic negative selection kit, StemCell Technologies, Vancouver, BC, Canada) and CD326 (EpCAM) Antibody, anti-mouse, Biotin (QC Testing: Mouse, Clone: caa7-9G8, Cat# 130-118-075, 1/50 dilution, Miltenyi Biotec B.V. & Co. KG, Bergisch Gladbach, Germany). Assessment of the EasySep™ cell separation efficiency was carried out using flow cytometry as described above.

### 4.11. Cultivation of CD326^+^ Lung Epithelial Cells

Lung epithelial cells were cultured using as described in [51,52]. The fraction of CD326^+^ cells (10^6^ cells /1 mL of medium) obtained by magnetic sorting was cultured on collagen-coated plastic plates for T-25 cell cultures in 1:1 Hams F12: M199 (Sigma-Aldrich, USA) supplemented with 5% FBS (Sigma-Aldrich, St. Louis, MO, USA), 2 mM L-glutamine (Sigma-Aldrich, St. Louis, MO, USA), 100 U/mL penicillin and 100 μg/mL streptomycin (Sigma-Aldrich, St. Louis, MO, USA). After 24 h, the medium was changed to serum-free medium composed of 1:1 Hams F12: M199 (Sigma-Aldrich, St. Louis, MO, USA), 2 mM L-glutamine, 100 U/mL penicillin, 100 μg/mL streptomycin, 100 μg/mL insulin-transferrin-selenium (Sigma-Aldrich, St. Louis, MO, USA), 100 ng/mL hydrocortisone (Sigma-Aldrich, USA), and 10 ng/mL epidermal growth factor (Sigma-Aldrich, St. Louis, MO, USA). The medium was changed every one to two days. After a five-day culture cycle, CD326^+^ cells of groups 1 and 2 were detached from the plate using a warm 0.05% trypsin-EDTA solution (Sigma-Aldrich, St. Louis, MO, USA) and the medium was changed to Hams F12:M199 (1:1) 5% FBS (Sigma-Aldrich, St. Louis, MO, USA), 10 ng/mL epidermal growth factor (Sigma-Aldrich, St. Louis, MO, USA), 10 ng/mL basic fibroblast growth factor, 4 μg/mL heparin, 100 U/mL penicillin, 100 μg/mL streptomycin (Sigma-Aldrich, St. Louis, MO, USA). Cells were plated at a concentration of 3 × 10^5^ cells/1 mL on collagen-coated plastic T-25 plates. Treamid was used at a concentration of 10^−7^ M). The effects of Treamid on CD326^+^ cell culture were assessed by determining the levels of CFSE, CD49f, and apoptosis levels using a Cytation 3 Cell Imaging multimode reader (BioTek Instruments, Inc., Winooski, VT, USA)

### 4.12. Cellular Imaging

Images of lung CD31^+^ endothelial and CD326^+^ epithelial cells were obtained using Cytation 3 equipped with DAPI, green fluorescent protein (GFP) and Texas Red light cubes.

At the end of the incubation period, lung endothelial and epithelial cells were stained with Hoechst 33342, CFSE BD Horizon, Annexin V-iFluor 350, and 7-AAD. In addition, a CD49f -FITC antibody was used for the analysis of CD326^+^ epithelial cells. Images were taken on Cytation 3 (magnification of 4× or 20×) followed by cell analysis using Gen5™ data analysis software (Biotek, Bad Friedrichshall, Germany).

All images were preprocessed to align the background before performing the analysis.

### 4.13. Statistical Analysis

Statistical analysis was performed using SPSS (version 15.0, SPSS Inc., Chicago, IL, USA). Data were analyzed and presented as means ± standard error of the mean. Statistical significance was evaluated by Student’s *t*-test (for parametric data), or Mann–Whitney test (for nonparametric data) when appropriate. A *p*-value of less than 0.05 (by two-tailed testing) was considered an indicator of statistical significance.

## 5. Conclusions

Our study provided evidence that Treamid has anti-inflammatory and antifibrotic effects and induces lung tissue regeneration in animals with pulmonary fibrosis. The positive effect of treatment can be explained by a decrease in inflammation, a rupture of the causal relationship between inflammation and fibrosis, which reduces the activity of synthesis and deposition of collagen, and by a stimulation of endothelial progenitor cells. Our results suggest that Treamid may be a promising therapeutic agent for antifibrotic therapy and restoration of lung structure and function in IPF patients.

## Supplementary Materials

Supplementary materials can be found at https://www.mdpi.com/1422-0067/21/21/8380/s1. Figure S1: Photomicrographs of left lung sections (middle pulmonary field) obtained from male C57BL/6 mice (d8). Figure S2: The level of interleukin 13 in lung homogenate of male C57BL/6 mice on d21. Figure S3: The content of epithelial cells (CD45^−^Ter119^−^CD326^+^) isolated from the lungs of C57BL/6 mice on d21. Figure S4: Effects of Treamid on CD326^+^ epithelial cell culture isolated from the lungs of male C57BL/6 mice. Figure S5: Content of interstitial macrophages (CD45^+^CD19^−^Sca1^+^CD11b^+^F4/80^+^) isolated from the lungs of C57BL/6 mice on d21. Figure S6: Content of MSC (CD45^‒^CD31^‒^CD34^‒^CD73^+^CD90^+^) (% of all stained mononuclear cells) and expression of the Notch1 in lung MSCs of male C57BL/6 mice on d21. Figure S7: Relative content (% of the total number of lung cells) of specific markers CD31 (a) and CD16 (b) in the lungs of male C57BL/6 mice. Figure S8: Content of the total population of mononuclear phagocytes CD11b+, alveolar macrophages (CD45^+^CD19^−^Sca1^+^CD11b^−^F4/80^+^) and circulating monocytes and lymphocytes (CD45^+^CD90^+^CD44^+^) isolated from the lungs of C57BL/6 mice on d21.

## Abbreviations

IPF	Idiopathic pulmonary fibrosis
MSC	Mesenchymal stem cells
BDDA	Bisamide derivative of dicarboxylic acid or Treamid
BLM	Bleomycin
Lm	Mean linear intercept
DI	Destructive index
PBS	Phosphate buffered saline
FBS	Fetal bovine serum
IL 13	Interleukin 13
CFSE	Carboxyfluorescein succinimidyl ester
