# Supplementary material for: Antifibrotic and Regenerative Effects of Treamid in Pulmonary Fibrosis
Source: Int J Mol Sci. 2020 Nov 8;21(21):8380. doi: 10.3390/ijms21218380 (PMC7664690; doi:10.3390/ijms21218380)
Supplement: Supplementary file 1 [file ijms-21-08380-s001.pdf]

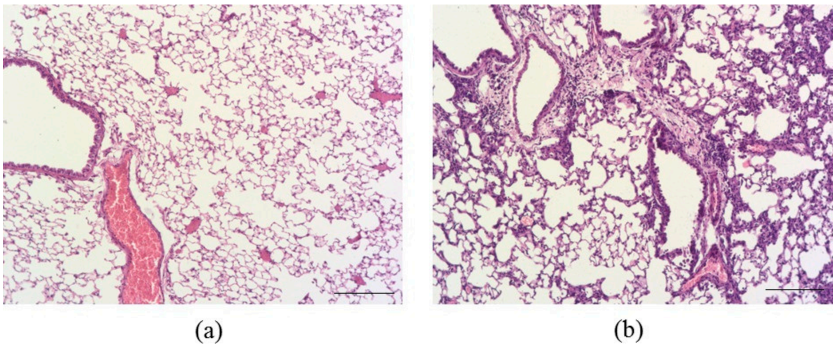

Figure S1. Photomicrographs of left lung sections (middle pulmonary field) obtained from male C57BL/6 mice (d8). (a) Intact control; (b) mice with pulmonary fibrosis, scale bar 100μm.

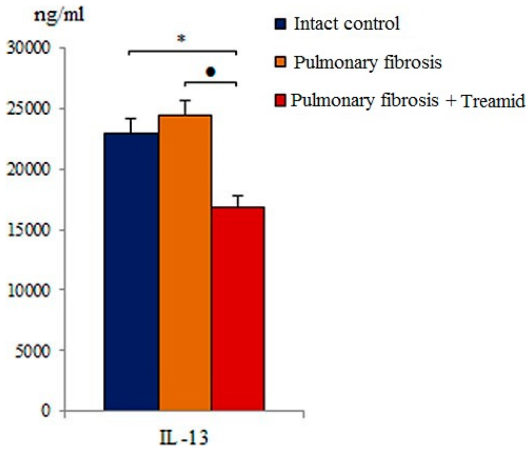

Figure S2. The level of interleukin 13 in lung homogenate of male C57BL/6 mice on d21. Groups: Intact control; mice with pulmonary fibrosis (Pulmonary fibrosis); mice with pulmonary fibrosis treated Treamid (Pulmonary fibrosis + Treamid). * – $p<0.05$ significance of difference compared with intact control group, ● $p<0.05$ significance of difference compared with the Pulmonary fibrosis group.

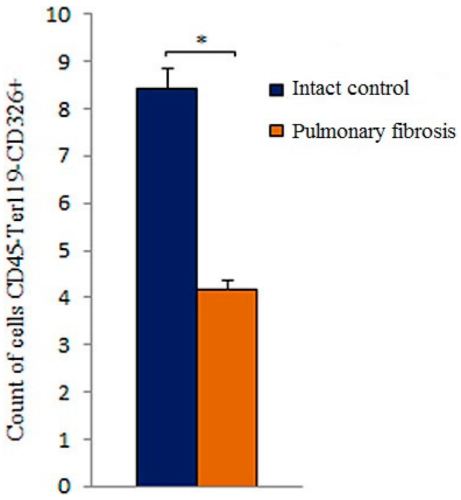

Figure S3. The content of epithelial cells ($CD45^{-}Ter119^{-}CD326^{+}$) isolated from the lungs of C57BL/6 mice on d21. Groups: intact control and pulmonary fibrosis. Cells were analyzed by flow cytometry using antibodies for mice, CD45, Ter119, and CD326. Dot plots are representative of three independent experiments with the mean of three independent experiments. * – $p<0.05$ significance of difference compared with intact control group.

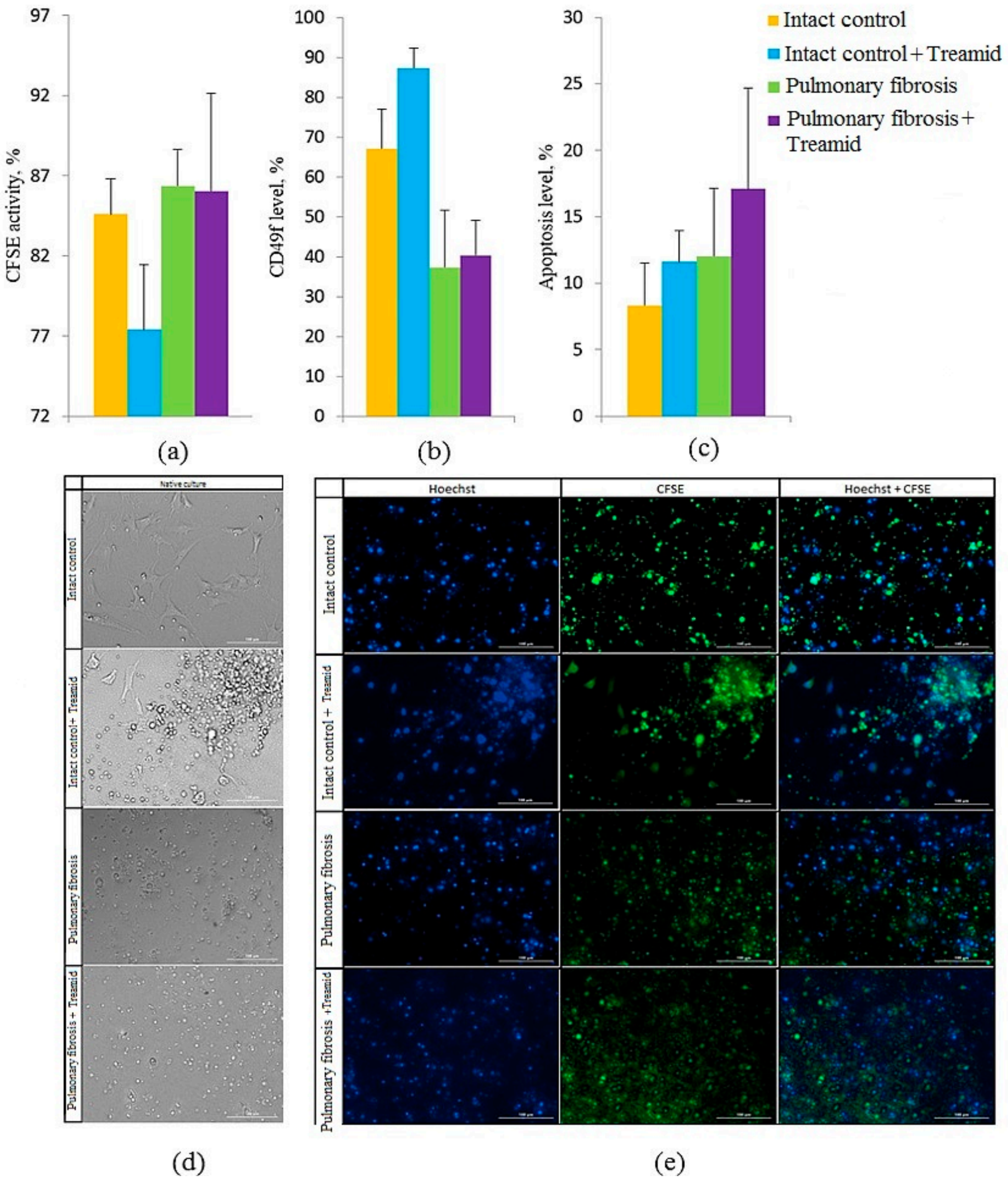

Figure S4. Effects of Treamid on $CD326^+$ epithelial cell culture isolated from the lungs of male C57BL/6 mice. (**a**) The level of CFSE activity, (**b**) the number of CD49f cells, and (**c**) cells in apoptosis in culture of $CD326^+$ lung cells from male C57BL/6 mice on day 5 of cell culture with or without Treamid (10^{-7} M). Treamid was added on the 5th day of culture for 1 hour. All data are expressed as mean ± SD. (**d**) Culture of $CD326^+$ lung epithelial cells was precultured for 5 days, incubated with or without Treamid (10^{-7} M) for 1 h; (**e**) Culture of $CD326^+$ lung epithelial cells was precultured for 5 days, incubated with or without Treamid (10^{-7} M) for 1 h and then labeled with Hoechst and CFSE (FITC) prior to fluorescence microscopic analysis. 20× images of $CD326^+$ lung cells stained with: Hoechst (blue) to identify cell nuclei; CFSE (green); (Hoechst + CFSE) composite image using all two colors. Determination of the percent of cells with CFSE activity is made by the ratio of cells counted in green channel to total cells counted using blue (DAPI) channel. All scale bars are 100 μm.

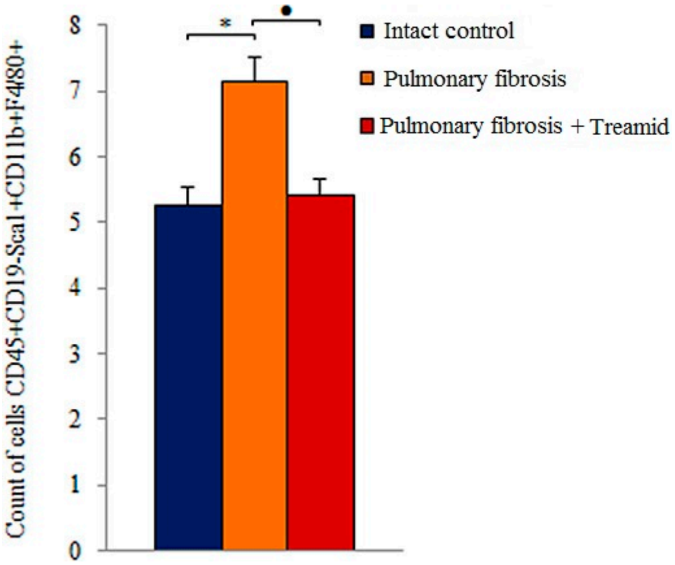

Figure S5. Content of interstitial macrophages ($CD45^{+}CD19^{-}Sca1^{+}CD11b^{+}F4/80^{+}$) isolated from the lungs of C57BL/6 mice on d21. Groups: Intact control; mice with pulmonary fibrosis (Pulmonary fibrosis); mice with pulmonary fibrosis treated Treamid (Pulmonary fibrosis + Treamid). The cells were analyzed by flow cytometry using antibodies for mice CD11b, CD19, CD45, Sca1, F4/80. Dot plots are representative of three independent experiments with the mean of three independent experiments. * – $p<0.05$ significance of difference compared with intact control group, ● $p<0.05$ significance of difference compared with the Pulmonary fibrosis group.

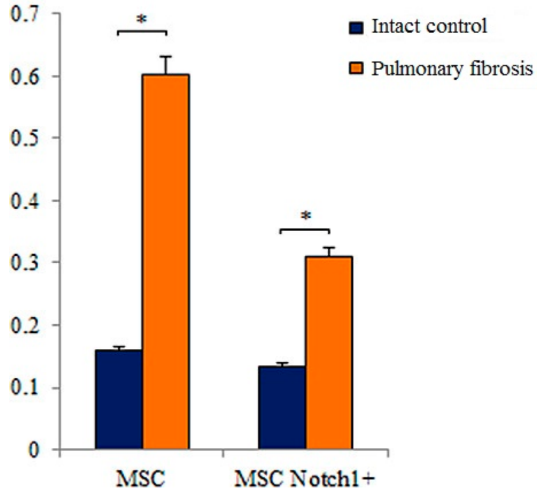

Figure S6. Content of MSC ($CD45^{-}CD31^{-}CD34^{-}CD73^{+}CD90^{+}$) (% of all stained mononuclear cells) and expression of the Notch1 in lung MSCs of male C57BL/6 mice on d21. Groups: Intact control; mice with pulmonary fibrosis (Pulmonary fibrosis). * – $p<0.05$ significance of difference compared with intact control group.

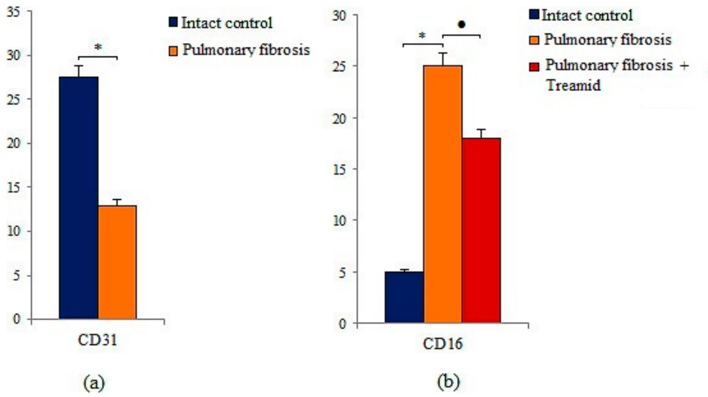

Figure S7. Relative content (% of the total number of lung cells) of specific markers CD31 (**a**) and CD16 (**b**) in the lungs of male C57BL/6 mice. Immunohistochemical staining for specific cellular markers: CD16 and CD31 in the lungs of mice was performed on d21. Lung tissue sections were placed on glass slides with an adhesive poly-lysine coating (Leica biosystems, Wetzlar, Germany). Prior to the staining, tissue sections were dewaxed, followed by antigen unmasking in citrate buffer (pH = 6) for 20 minutes. Incubation with primary antibodies was performed in a humidity chamber at 37° C. To detect specific cell markers, the following primary antibodies were used: rabbit polyclonal anti CD16 (ab203883, 1/100, species reactivity: Mouse, Rat, Human, Abcam, USA) and rabbit polyclonal anti CD31 (ab28364, 1/50, species reactivity: Mouse, Human, Pig, Abcam, USA). The sections were contrasted with hematoxylin. Visualization system was used in accordance with the manufacturer's instructions for antibody detection (Spring bioscience, USA). After staining, the sections were dehydrogenated in xylene and enclosed in a mounting medium (Leica CV Ultra, Cat# 14070937891, Leica Biosystems, Wetzlar, Germany). An Axio Lab.A1 microscope (Carl Zeiss, Germany) with an AxioCam ERc5s camera (Carl Zeiss, Germany) was used to obtain micrographs. Image analysis and calculation of cells expressing detectable antigens were performed with FIJI. Groups: intact control – control group from intact mice; mice with pulmonary fibrosis (Pulmonary fibrosis); mice with pulmonary fibrosis treated Treamid (Pulmonary fibrosis + Treamid). * – $p<0.05$ significance of difference compared with intact control group, ● $p<0.05$ significance of difference compared with the Pulmonary fibrosis group.

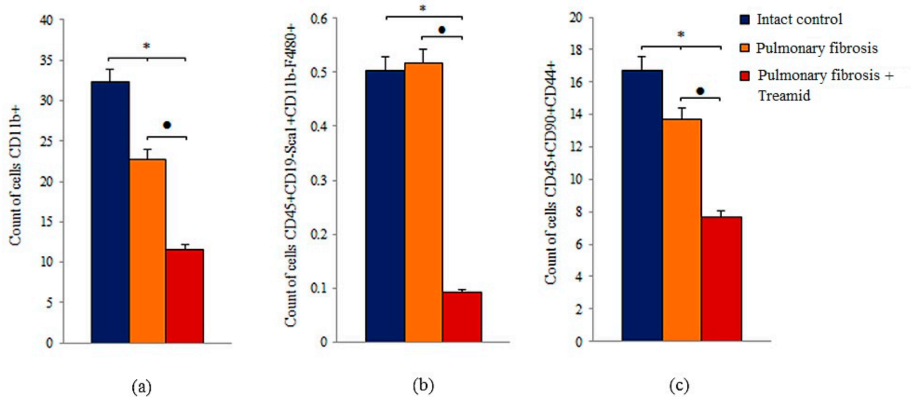

Figure S8. Content of the total population of mononuclear phagocytes $CD11b^{+}$, alveolar macrophages ($CD45^{+}CD19^{-}Sca1^{+}CD11b^{-}F4/80^{+}$) and circulating monocytes and lymphocytes ($CD45^{+}CD90^{+}CD44^{+}$) isolated from the lungs of C57BL/6 mice on d21. The cells were analyzed by flow cytometry using antibodies for mice CD11b, CD19, CD44, CD45, CD90, Sca1, F4/80. Dot plots are representative of three independent experiments with the mean of three independent experiments. Groups: Intact control; mice with pulmonary fibrosis (Pulmonary fibrosis); mice with pulmonary fibrosis treated Treamid (Pulmonary fibrosis +Treamid). (**a**) Cell counts of the general population of mononuclear phagocytes ($CD11b^{+}$) in the lungs of mice; (**b**) the content of alveolar macrophages ($CD45^{+}CD19^{-}Sca1^{+}CD11b^{-}F4/80^{+}$) in the lungs of mice; (**c**) the content of circulating monocytes and lymphocytes ($CD45^{+}CD90^{+}CD44^{+}$) in the lungs of mice. * – $p<0.05$ significance of difference compared with intact control group, ● $p<0.05$ significance of difference compared with the Pulmonary fibrosis group.
